# Tricarboxylic Acid Cycle and One-Carbon Metabolism Pathways Are Important in *Edwardsiella ictaluri* Virulence

**DOI:** 10.1371/journal.pone.0065973

**Published:** 2013-06-07

**Authors:** Neeti Dahal, Hossam Abdelhamed, Jingjun Lu, Attila Karsi, Mark L. Lawrence

**Affiliations:** 1 Department of Basic Sciences, College of Veterinary Medicine Sciences, Mississippi State University, Mississippi State, Mississippi, United States of America; 2 Department of Fish Diseases and Management, Faculty of Veterinary Medicine, Benha University, Moshtohor-Toukh, Egypt; Auburn University, United States of America

## Abstract

*Edwardsiella ictaluri* is a Gram-negative facultative intracellular pathogen causing enteric septicemia of channel catfish (ESC). The disease causes considerable economic losses in the commercial catfish industry in the United States. Although antibiotics are used as feed additive, vaccination is a better alternative for prevention of the disease. Here we report the development and characterization of novel live attenuated *E. ictaluri* mutants. To accomplish this, several tricarboxylic acid cycle (*sdhC*, *mdh*, and *frdA*) and one-carbon metabolism genes (*gcvP* and *glyA*) were deleted in wild type *E. ictaluri* strain 93-146 by allelic exchange. Following bioluminescence tagging of the *E. ictaluri* Δ*sdhC*, Δ*mdh*, Δ*frdA*, Δ*gcvP*, and Δ*glyA* mutants, their dissemination, attenuation, and vaccine efficacy were determined in catfish fingerlings by in vivo imaging technology. Immunogenicity of each mutant was also determined in catfish fingerlings. Results indicated that all of the *E. ictaluri* mutants were attenuated significantly in catfish compared to the parent strain as evidenced by 2,265-fold average reduction in bioluminescence signal from all the mutants at 144 h post-infection. Catfish immunized with the *E. ictaluri* Δ*sdhC*, Δ*mdh*, Δ*frdA*, and Δ*glyA* mutants had 100% relative percent survival (RPS), while *E. ictaluri* Δ*gcvP* vaccinated catfish had 31.23% RPS after re-challenge with the wild type *E. ictaluri*.

## Introduction

Channel catfish, *Ictalurus punctatus*, farming is the largest aquaculture industry in the United States, and enteric septicemia of catfish (ESC), caused by *Edwardsiella ictaluri*, is the most prevalent disease affecting this industry. Although Romet® 30, Terramycin®, and Aquaflor® are approved antibiotics to treat infections in commercial catfish by oral delivery in medicated feed, effectiveness is limited because fish develop anorexia at early stages of the infection. Also, antibiotic resistant *E. ictaluri* strains can emerge [1]. Therefore, vaccination is the preferred method for prevention of ESC.

Live attenuated vaccines can provide effective protection against certain diseases if they can express protective antigens without causing disease in the host [2]. In *E. ictaluri*, some candidate live attenuated vaccines that have been developed include chondroitinase [3] and auxotrophic (*aroA* and *purA*) [4], [5] mutants. However, none of these vaccine candidates are in commercial production. The commercial vaccine Aquavac-ESC (RE-33) was developed by selecting for rifampin resistance [6]. However, antibiotic resistance is not a desired trait for a vaccine. In addition, the genetic basis for attenuation in RE-33 is undefined [7], although it is known that RE-33 expresses shortened LPS O side chains [8]. Despite the availability of Aquavac-ESC, ESC is still the most prevalent disease in the catfish industry [9], [10].

*E. ictaluri* is considered a facultative intracellular pathogen, and it is capable of surviving inside channel catfish neutrophils and macrophages [11], [12]. Although *E. ictaluri* is effectively phagocytosed by catfish neutrophils, it is only killed by neutrophils to a limited extent [11], [13]. A recent study by Karsi et al. [14] showed that genes encoding tricarboxylic acid (TCA) cycle enzymes, glycine cleavage system, a sigmaE regulator, the SoxS oxidative response system, and a plasmid-encoded type III secretion system (TTSS) effector are important for survival in neutrophils [14]. The same study discovered that some neutrophil-susceptible *E. ictaluri* strains were highly attenuated and demonstrated very good potential as live attenuated vaccines. In particular, strains with insertion mutations in genes encoding TCA cycle enzymes succinate dehydrogenase (*sdhC*) (*Ei*AKMut5) and malate dehydrogenase (*mdh*) (*Ei*AKMut12) generated better protection than the available commercial vaccine when juvenile catfish were vaccinated by immersion [14]. Similarly, *E. ictaluri* glycine dehydrogenase (*gcvP*) mutants (*Ei*AKMut02 and *Ei*AKMut08) were also completely attenuated and had better vaccine efficacy than the commercial vaccine [14]. Glycine dehydrogenase is part of the glycine cleavage system pathway, which is part of one-carbon (C1) metabolism. Therefore, the objective of this research was to introduce in-frame deletions in *E. ictaluri sdhC*, *mdh*, and *frdA* genes (encoding enzymes in the TCA cycle) and *gcvP* and *glyA* genes (encoding C1 metabolism proteins) to determine their roles in *E. ictaluri* virulence.

## Materials and Methods

### Ethics statement

All fish experiments were conducted in accordance with a protocol approved by the Institutional Animal Care and Use Committee (IACUC) at Mississippi State University.

### Bacterial strains, plasmids, and growth conditions

Bacterial strains and plasmids used in this work are listed in Table 1. *E. ictaluri* was grown at 30°C using brain heart infusion (BHI) broth and agar (Difco, Sparks, MD). *Escherichia coli* were grown at 37°C using Luria-Bertani (LB) broth and agar (Difco). *E. coli* CC118 λ*pir* and SM10 λ*pir*/S17-1 λ*pir* were used for cloning gene deletions into suicide plasmid pMEG-375 and transferring recombinant pMEG-375 or pAK*gfplux*1 into *E. ictaluri*. Ampicillin was used at 100 µg/ml to maintain pMEG-375 and pAK*gfplux*1. Colistin was used at 12.5 µg/ml for counter selection against *E. coli* SM10 λ*pir* following conjugation. *E. ictaluri* strains were cultivated for 18 h (stationary phase) for all fish challenges.

**Table 1:** Bacterial strains and plasmids.

Strain	Relevant Characteristics	References
*Edwardsiella ictaluri*		
93-146	Wild type; pEI1^+^; pEI2^+^; Col^r^	[32]
*Ei*Δ*frdA*	93-146 derivative; pEI1^+^; pEI2^+^; Col^r^; Δ*frdA*	This study
*Ei*Δ*gcvp*	93-146 derivative; pEI1^+^; pEI2^+^; Col^r^; Δ*gcvP*	This study
*Ei*Δ*glyA*	93-146 derivative; pEI1^+^; pEI2^+^; Col^r^; Δ*glyA*	This study
*Ei*Δ*sdhC*	93-146 derivative; pEI1^+^; pEI2^+^; Col^r^; Δ*sdhC*	This study
*Ei*Δ*mdh*	93-146 derivative; pEI1^+^; pEI2^+^; Col^r^; Δ*mdh*	This study
*Escherichia coli*		
CC118 λ*pir*	Δ(*ara*-*leu*); *araD*; Δ*lacX74*; *galE*; *galK*; *phoA20*; *thi-1*; *rpsE*; *rpoB*; *argE*(Am); *recAl*; λ*pir*R6K	[33]
SM10 λ*pir*	*thi*; *thr*; *leu*; *tonA*; *lacY*; *supE*; *recA*; ::RP4-2-Tc::Mu; Km^r^; λ*pir*R6K	[34]
S17-1 λ*pir*	*RP4-2 (Km::Tn7, Tc::Mu-1), ΔuidA3::pir^+^, recA1, endA1, thi-1,hsdR17, creC510*	[35]
Plasmids		
pMEG-375	8142 bp, Amp^r^, Cm^r^, *lacZ*, R6K *ori*, *mob incP*, *sacR sacB*	[36]
p*Ei*Δ*frdA*	10242 bp, Δ*frdA*, pMEG-375	This study
p*Ei*Δ*gcvp*	12231 bp, Δ*gcvP*, pMEG-375	This study
p*Ei*Δ*glyA*	14276 bp, Δ*glyA*, pMEG-375	This study
p*Ei*Δ*sdhC*	16295 bp, Δ*sdhC*, pMEG-375	This study
p*Ei*Δ*mdh*	18350 bp, Δ*mdh*, pMEG-375	This study

### Construction and bioluminescence tagging of in-frame deletion mutants

The method of overlap extension PCR [15] was used to generate in-frame deletions of *E. ictaluri sdhC*, *mdh*, *frdA*, *gcvP*, and *glyA*. Four primers were designed for each gene including forward (lflp), internal-reverse (lfrp), internal forward (rflp), and reverse primers (rfrp) (Table 2). Restriction sites were included in forward and reverse primers. Genomic DNA was isolated from *E. ictaluri* using a Wizard Genomic DNA Kit (Promega, Madison, WI) and used as template in PCR. Upper fragments were amplified by forward and internal-reverse primer sets while reverse and internal-forward primer sets were used to amplify lower fragments. The resulting upper and lower PCR products were gel extracted using a QIAquick Gel Extraction Kit (Qiagen, Valencia, CA), mixed in a 1∶1 ratio, and then re-amplified using the forward and reverse primers. The resulting in-frame deleted fragment was purified by using a QIAquick Gel Extraction Kit (Qiagen, Valencia, CA). The purified PCR product was digested with appropriate restriction enzymes (Promega) (Table 1) and cleaned using a Wizard SV Gel and PCR Clean-Up Kit (Promega).

**Table 2:** Primers with restriction enzyme used for the construction of the *E. ictaluri* mutants.

Genes	Primer ID	Primer Sequence ((5′→3′)^a^	RE^b^
*frdA*	*EifrdA*lflp	AA**GAGCTC**TCGTCCACTTCATTCATCAGAC	*Sac*I
	*EifrdA*lfrp	GTGGAAGTGGAAATCGAAAGA	
	*EifrdA*rflp	TCTTTCGATTTCCACTTCCACGAAGCTCAGGAAGCCAAGAAG	
	*EifrdA*rfrp	AA**TCTAGA**GCAGGGAGATGATATTGAGGAC	*Xba*I
	*EifrdA*01S	CCTCAACTGAAGATTGCCTTA	
*gcvP*	*EigcvP*lflp	AA**TCTAGA**CCTTTGGCGTGGAGATATGC	*Xba*I
	*EigcvP*lfrp	AGCATCACTGTTTTCAAGCTG	
	*EigcvP*rflp	CAGCTTGAAAACAGTGATGCTGTAAAGCGCCTGGACGATGT	
	*EigcvP*rfrp	AA**GAGCTC**CGGACAGAGACATACCACCAA	*Sac*I
	*Eigcvp*01S	GGCCTTTTGGTATGATTTGC	
*glyA*	*EiglyA*lflp	AA**GAGCTC**GGGCATGGGTCAGTGAATAC	*Sac*I
	*EiglyA*lfrp	CCACAGCTCGGTATCGTAATC	
	*EiglyA*rflp	GATTACGATACCGAGCTGTGGGTGAACGTCTTCCGGTCTATG	
	*EiglyA*rfrp	AA**CCCGGG**GCCTAGACGATGTCTCCTTGA	*Sma*I
	*EiglyA*01S	GGGCCAGATTTACTCAAAACC	
*sdhC*	*EisdhC*lflp	AA**GAGCTC**CAGCCTCCTTTGGTACTGCTA	*Sac*I
	*EisdhC*lfrp	GCAAATCCAGATTGACAGGTCT	
	*EisdhC*rflp	AGACCTGTCAATCTGGATTTGCGGGTATGGTAAGCAACGCATC	
	*EisdhC*rfrp	AA**CCCGGG**CCCCATCATGTAGTGACAGGT	*Sma*I
	*EisdhC*01S	CTCAGTCTCGTGGGATTTGC	
*mdh*	*Eimdh*lflp	AA**GAGCTC**GGCTTTATAATGGCGTGTGG	*Sac*I
	*Eimdh*lfrp	AGGCAGCTGAGTCTTAAGCAG	
	*Eimdh*rflp	CTGCTTAAGACTCAGCTGCCTCTGGGCGAAGACTTTATCAAT	
	*Eimdh*rfrp	AA**CCCGGG**GGAGCAGGCCCTACAAGACT	*Sma*I
	*Eimdh*01S	CAGCTCGCAATCTGAGTGTT	

a RE: restriction enzyme sequence added to the 5′ end of the primer sequence.

b Bold letters at the 5′ end of the primer sequence represent RE site. AA nucleotides were added to the end of each primer containing a RE site to increase the efficiency of enzyme cut. Underlined bases in internal primer (rflp) indicate reverse complemented internal primer (lfrp) sequence.

The suicide plasmid pMEG-375 was purified from an overnight *E. coli* culture by a QIAprep Spin Miniprep Kit (Qiagen) and cut with restriction enzymes respective to the inserts, producing compatible ends. The purified PCR product with in-frame deletion was ligated into pMEG-375 vector using T4 DNA Ligase (Promega) at 4°C overnight, generating p*EiΔsdhC*, p*EiΔmdh*, p*EiΔfrdA*, p*EiΔgcvP*, and p*EiΔglyA* (Table 1). Insert in each plasmid was confirmed by restriction enzyme digestion as well as sequencing.

The suicide plasmids with in-frame deleted genes were transferred into *E. coli* SM10 λpir/S17-1 λ*pir* by electroporation and mobilized into *E. ictaluri* 93-146 by conjugation [16]. The recipient bacteria were spread on BHI plates containing colistin (12.5 ug/ml) and ampicillin (100 ug/ml) to select *E. ictaluri* with integrated vector by single crossover through allelic exchange. Ampicillin resistant colonies were propagated on BHI plates to allow for the second crossover allelic exchange, followed by streaking on BHI plates with 5% sucrose, 0.35% mannitol, and colistin to select for loss of pMEG-375 with *sacB* gene. Potential mutant colonies were tested for ampicillin sensitivity to ensure loss of the plasmid. Deleted regions were amplified from the resulting ampicillin sensitive colonies and confirmed by sequencing. After confirmation, *Ei*Δ*sdhC*, *Ei*Δ*mdh*, *Ei*Δ*frdA*, *EiΔgcvP*, and *Ei*Δ*glyA* mutants were labeled with bioluminescence using pAK*gfplux*1 as described in Karsi and Lawrence [16].

### Mutant virulence and ability to protect against *E. ictaluri* infection

Experimental infections were conducted in 40-L challenge tanks supplied with flow-through dechlorinated municipal water. Water temperature was maintained at 25°C (±2) throughout the experiments. Twenty-eight specific pathogen free (SPF) catfish fingerlings (14.2±0.35 cm, 25.45±1.82 g) were randomly allocated into seven groups (4 fish/group). Five treatments were injected with *E. ictaluri* mutants, one group was injected with wild type *E. ictaluri* strain 93-146, and the last group served as negative control (phosphate-buffered saline (PBS). Fish were anesthetized in water containing 100 mg/L MS222 and injected with approximately 1×10^4^ colony forming units (CFU) in 100 µl PBS.

Bioluminescent imaging (BLI) was conducted using an IVIS 100 Imaging System to measure number of photons emitted by bioluminescent bacteria in fish [17]. Briefly, catfish were anesthetized in water containing 100 mg/L MS222 and transferred immediately to the photon collection chamber for image capture. Total photon emissions from the whole fish body were collected at an exposure time of one min. Following BLI imaging, fish were returned to well-aerated water for recovery. BLI was conducted at 2, 4, 8, and 24 h post-infection, and subsequent daily intervals until 168 h. Bioluminescence was quantified from the fish images using Living Image Software v 2.5 (Caliper Corporation., Hopkinton, Massachusetts), and mean photon counts for each treatment were used in statistical analysis.

To determine the ability of mutants to protect against *E. ictaluri* infection, the juvenile catfish vaccinated with mutants (virulence challenge) were immersion challenged [14] with 4.8×10^7^ bioluminescent wild type *E. ictaluri* at 4 weeks post-vaccination. Photon emissions from fish were collected at 2, 4, 8, 24, 48, 72, and 96 h post-infection using an IVIS 100 as described above, and statistical analysis was performed on the mean photon counts.

### Mutant ability to protect against ESC induced mortalities

Approximately 420 eight-month-old SPF channel catfish fingerlings (17.61±0.63 cm, 47.47±5.31 g) were stocked into 21 tanks at a rate of 20 fish/tank. Each treatment had three replicate tanks. Treatments consisted of *Ei*Δ*sdhC*, *Ei*Δ*mdh*, *Ei*Δ*frdA*, *Ei*Δ*glyA and Ei*Δ*gcvP* (vaccination), wild type *E. ictaluri* (positive control), and BHI (sham control). Channel catfish were vaccinated by immersion in water containing approximately 4.3×10^7^ CFU/ml of water for 1 h, followed by gradual removal of bacteria. Mortalities were recorded for 21 days following vaccination. At 21 days post-vaccination, both vaccinated and non-vaccinated treatments were immersion exposed to wild type parent *E. ictaluri* 93-146 (approximately 3.06×10^7^ CFU/ml), and fish mortalities were recorded daily for 14 days. Relative percent survival (RPS) was calculated according to the following formula: RPS = [1−(% mortality of vaccinated fish/% mortality of non-vaccinated fish)]×100 [18].

### Statistical analysis

Photon counts were transformed by taking the base 10 logarithm to improve normality. One-way ANOVA was conducted using SPSS V19 (IBM Corp., Armonk, NY) to compare mean photon counts at each time point (*p*<0.05). Pairwise comparison of the means was done using Tukey procedure. Data was then retransformed for interpretation.

## Results

### Construction of the *E. ictaluri* in-frame deletion mutants

**Figure 1:**
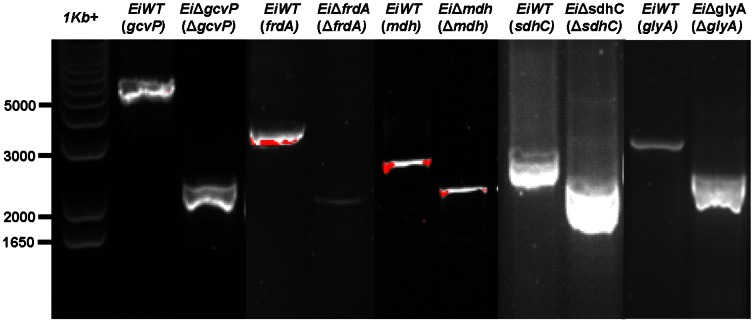
Genotypic confirmation of the *E. ictaluri gcvP*, *frdA*, *mdh*, *sdhC*, and *glyA* mutants. Genomic DNAs was amplified from the *E. ictaluri* wild type and mutants using the two outside primers (lflp and rfrp) and separated on 1% agarose gel.

Five in-frame mutants (*Ei*Δ*sdhC*, *Ei*Δ*mdh*, *Ei*Δ*frdA*, *EiΔgcvP*, and *Ei*Δ*glyA*) were obtained successfully (Fig. 1) by deleting on average over 90% of each gene (Table 3).

**Table 3:** Properties of the *E. ictaluri* TCA cycle and C1 metabolism genes and percentage of gene deleted.

Gene	Locus	Product	ORF (bp/aa)	Remaining (bp/aa)	% Deletion
*sdhC*	NT01EI_2872	Succinate dehydrogenase, cytochrome b556 subunit, putative	390/129	57/18	86.05
*mdh*	NT01EI_0446	Malate dehydrogenase, NAD-dependent, putative	939/312	99/32	89.74
*frdA*	NT01EI_0392	Fumarate reductase, flavoprotein subunit, putative	1800/899	126/41	95.44
*gcvP*	NT01EI_3351	Glycine dehydrogenase, putative	2884/960	114/37	96.15
*glyA*	NT01EI_3190	Serine hydroxymethyltransferase, putative	1254/417	75/24	94.24

### Mutant virulence and ability to protect against *E. ictaluri* infection

**Figure 2:**
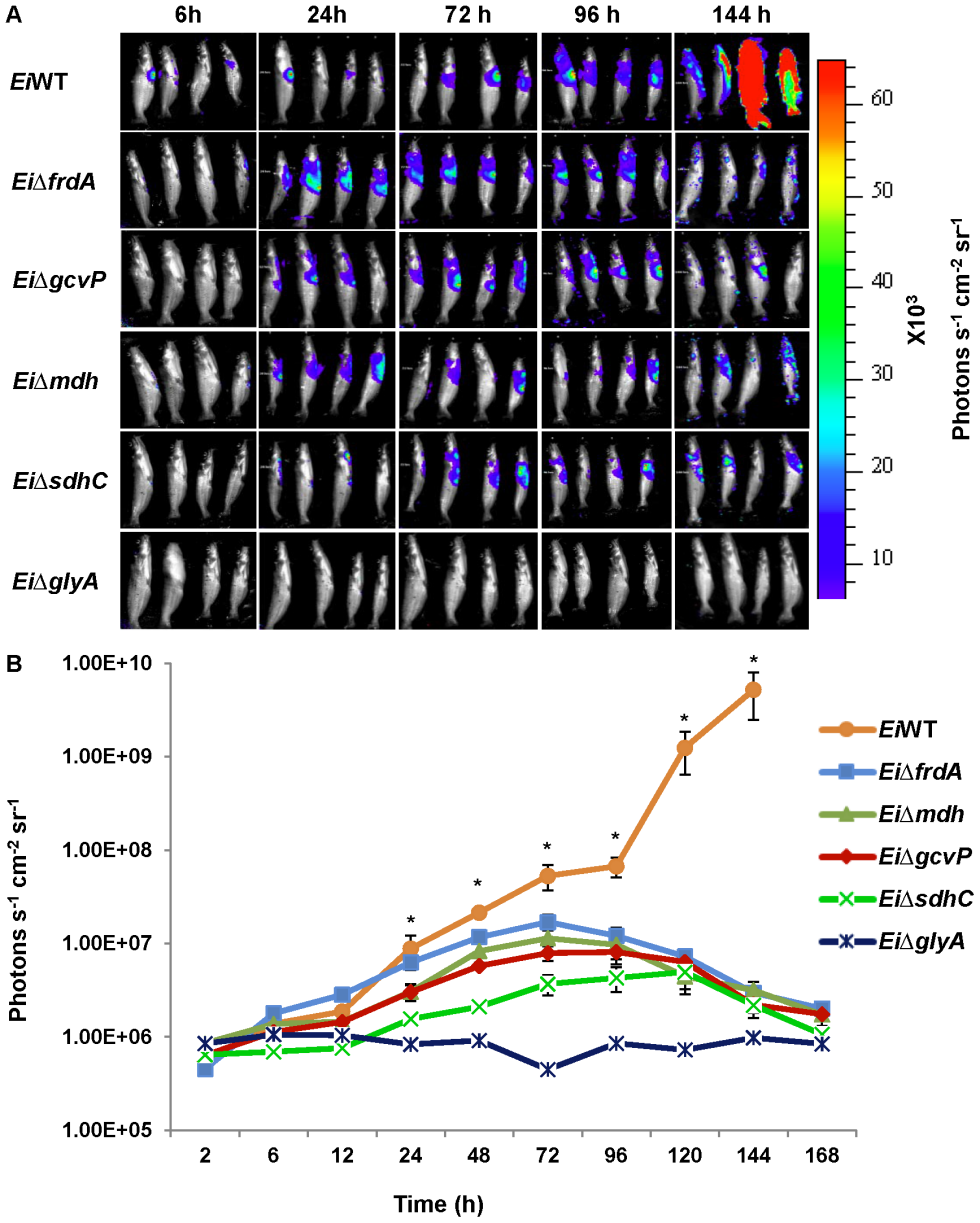
Bioluminescent imaging of vaccination/attenuation in live catfish after intraperitoneal injection. A, BLI imaging of catfish. B, Total photon emissions from each fish. Each data point represents the mean photon emissions from four fish. Two of the four channel catfish injected with wild type died at 144 h post-infection. The remaining two died at 168 h post-infection. Star indicates significant difference between wild type *E. ictaluri* and other mutants, except for *EiΔfrdA* at 24 h.

BLI results revealed that bioluminescence (quantified as photon counts) from the catfish infected with *Ei*Δ*sdhC*, *Ei*Δ*mdh*, *Ei*Δ*frdA*, *Ei*Δ*gcvP*, and *Ei*Δ*glyA* mutants were low at 2, 6, and 12 h post-infection. However, bioluminescence for mutants *Ei*Δ*mdh*, *Ei*Δ*frdA*, *and Ei*Δ*gcvP* increased from 24 h to 72 h and then decreased thereafter. Bioluminescence for *Ei*Δ*sdhC* followed the same pattern as the other mutants, except the signal peaked at 120 h. However, in mutant *Ei*Δ*glyA*, very low bioluminescence was detected at all time points. In fish infected with wild type *E. ictaluri*, bioluminescence increased until all fish died (Fig. 2). Average photon counts in the fish infected with 93-146 at 72 h post-infection were approximately 7-fold higher than the average of all fish infected with mutant strains, and it was 2,265-fold higher at 144 h. At this time point, fish infected with wild type *E. ictaluri* strain died, while bioluminescence from fish infected with mutant strains was in decline (Fig. 2). Photon counts were 118- and 5,329-fold higher in wild type *E. ictaluri* compared to *Ei*Δ*glyA* at 72 h and 144 h post-infection, respectively (Fig. 2). In the wild type infected treatment, two fish died at 144 h post-infection, and the remaining two fish died at168 h. Mean photon counts between all mutants (except *Ei*Δ*frdA*) and wild type *E. ictaluri* were significantly different (*p*<0.5) at 24 h and thereafter. Mean photon counts for wild type *E. ictaluri* were significantly higher than *EiΔfrdA* at 48 h and thereafter.

**Figure 3:**
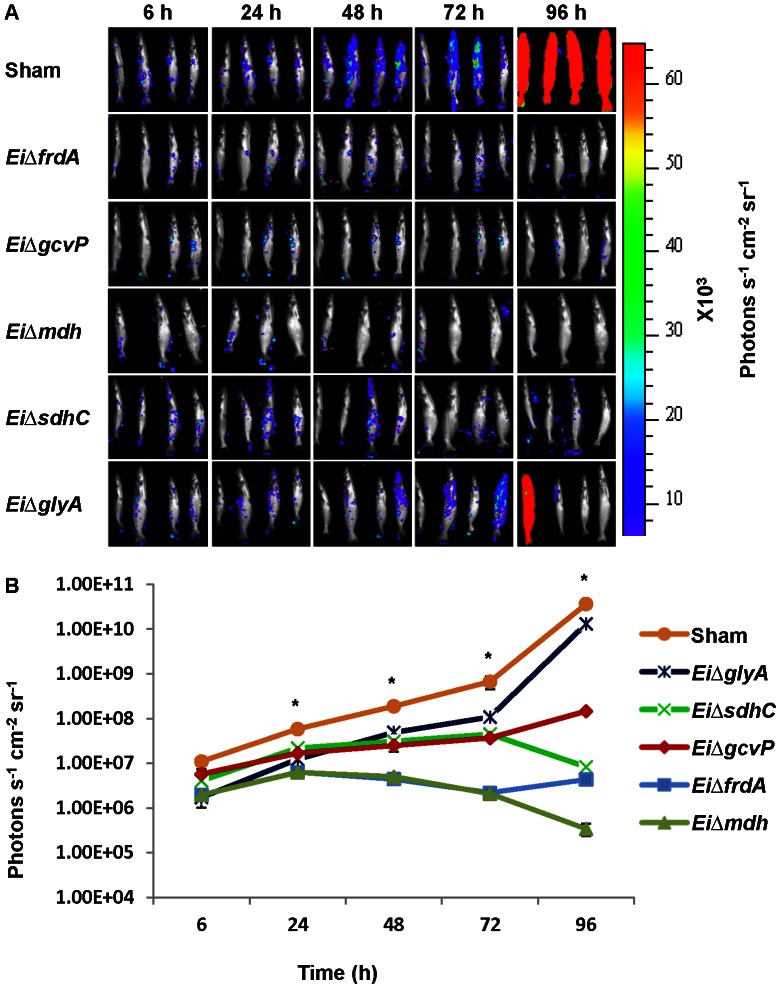
Bioluminescence imaging of juvenile catfish after immersion exposure to wild type *E. ictaluri*. Fish were challenged with *E. ictaluri* mutants as described in the virulence trial, and at 4 weeks post-vaccination they were challenged with bioluminescent wild type *E. ictaluri*. A, BLI imaging of catfish. B, Total photon emissions from each fish. Each data point represents the mean photon emissions from four fish. Star indicates significant difference between the *E. ictaluri* mutants and wild type.

When mutant challenged fish were immersion exposed to wild type *E. ictaluri* at 4 weeks post-vaccination, photon counts were significantly lower (*p*<0.5) at each time point for the vaccinated fish compared to the sham-vaccinated control (Fig. 3). Average photon counts in sham-vaccinated fish at 6 h post-infection were 4-fold higher than the average of all five mutant-vaccinated fish treatments, which increased to 14-fold at 96 h. At this time, bioluminescence in *Ei*Δ*sdhC* and *Ei*Δ*mdh* vaccinated fish was declining, while bioluminescence in *Ei*Δ*frdA*, *Ei*Δ*gcvP*, and *Ei*Δ*glyA* vaccinated fish was increasing (Fig. 3).At 96 h post-infection, all fish in the sham vaccinated group died. In summary, BLI demonstrated that all mutants are significantly attenuated compared to wild type *E. ictaluri*, and all mutants except *EiΔglyA* provided significant protection against *E. ictaluri* infection.

### Mutant ability to protect against ESC induced mortalities

**Figure 4:**
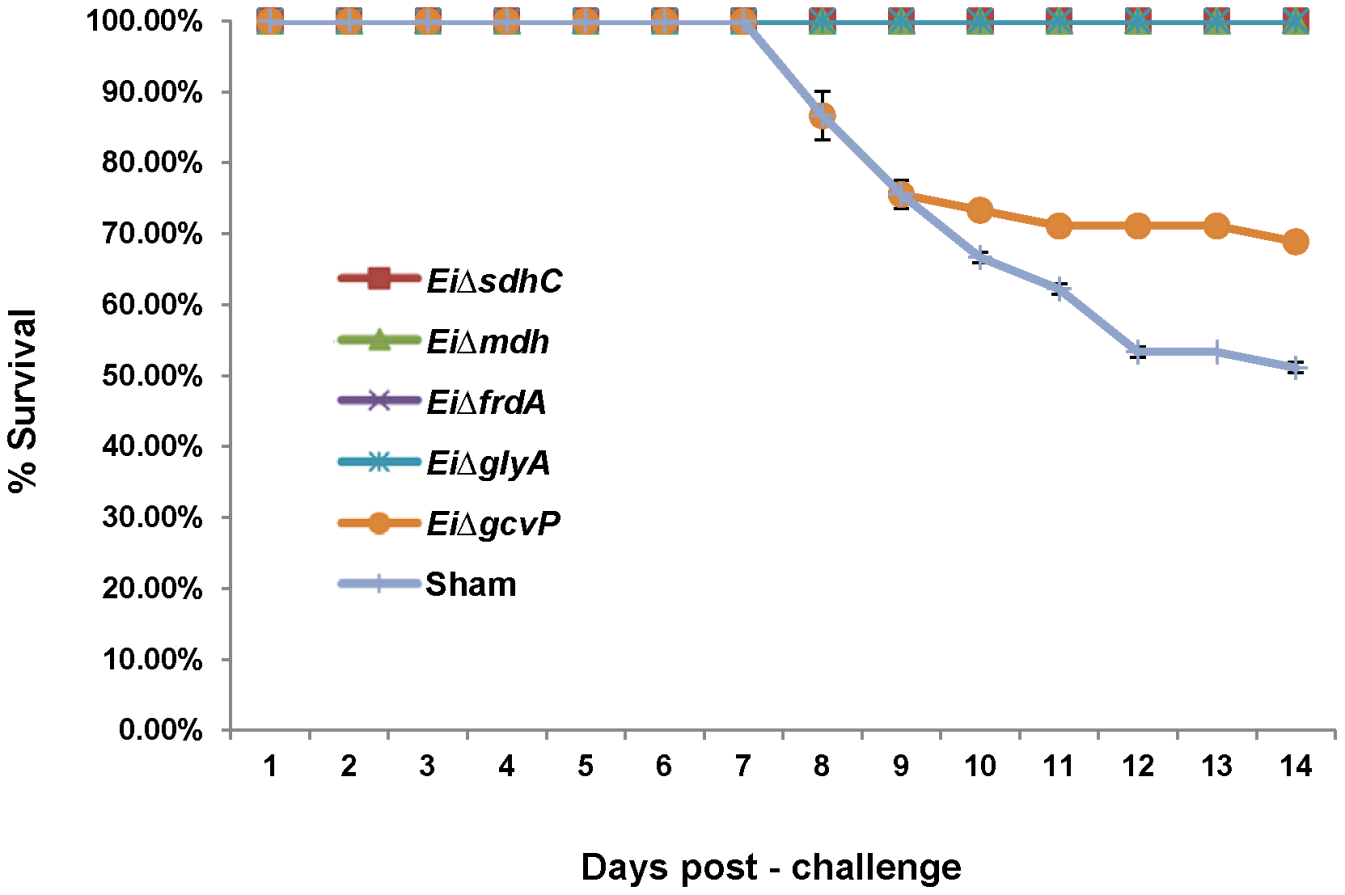
Percent survival of immersion vaccinated catfish. Catfish fingerlings were immersion vaccinated with the *EiΔmdh, EiΔsdhC, EiΔfrdA, EiΔglyA, and EiΔgcvP* mutants and challenged with wild type *E. ictaluri* strain 93-146. Data points represent the mean percent survival of 20 fish per tank for each treatment.

Vaccination of channel catfish with *Ei*Δ*sdhC*, *Ei*Δ*mdh*, *Ei*Δ*frdA*, and *Ei*Δ*glyA* provided complete protection (100% survival) against wild type *E. ictaluri* 93-146 while the *Ei*Δ*gcvP* mutant showed lower efficacy (68.89% survival) (Fig. 4). Survival in *Ei*Δ*sdhC*, *Ei*Δ*mdh*, *Ei*Δ*frdA*, and *Ei*Δ*glyA* vaccinated groups was 1.96-fold higher than that of the non-vaccinated group when re-challenged with wild type *E. ictaluri* (100% vs 51.11%).

## Discussion

The primary objective of this study was to construct live attenuated *E. ictaluri* strains based on mutations in genes encoding enzymes in the TCA cycle (*mdh*, *schC*, and *frdA*) and enzymes involved in C1 metabolism (*gcvP* and *glyA*). Additional aims included assessing the mutant strains' virulence in catfish and ability to protect against wild type *E. ictaluri* infection. We constructed in-frame deletion mutants to avoid polar effects of the mutations and to avoid insertion of antibiotic resistance genes, which is undesirable in vaccine strains. Splicing overlap extension combined with allelic exchange is an effective method for gene deletion in *E. ictaluri* and has been reported previously [19], [20].

We utilized bioluminescence imaging to assess virulence of mutants, which allows better quantification compared to percent mortalities. It also enables sensitive detection of subclinical infection and mutants' abilities to invade and establish infection. Mutant strains *Ei*Δ*sdhC*, *Ei*Δ*mdh*, *Ei*Δ*frdA*, and *Ei*Δ*gcvP* were clearly able to establish infection because bioluminescence was detected after 12 h post-infection. However, channel catfish injected with the mutant strains started clearing the bacteria after 72 h post-infection. Thus, our results showed that although *Ei*Δ*sdhC*, *Ei*Δ*mdh*, *Ei*Δ*frdA*, and *Ei*Δ*gcvP* do not cause mortalities, they are able to invade and establish infection before being cleared. Because of mutants' abilities to survive and replicate in fish up to 72 h post-infection, we expected them to generate an immune response and protection against wild type *E. ictaluri*. On the other hand, the *Ei*Δ*glyA* mutant did not replicate well in the host, and we anticipated much less systemic protection from this mutant. By contrast, wild type *E. ictaluri* increased in quantity until mortality occurred. Our current study corroborated an earlier study showing that 1×10^9^ photons^−1^ cm^−2^ steradian^−1^ seems to be a critical threshold for bacterial tissue concentrations where mortality is imminent [21].

Ultimately, prevention of mortalities is used as a common measure of vaccine efficacy. Thus, we used percent survival to evaluate efficacy of our candidate vaccines in catfish fingerlings using immersion exposure, which is a practical route of vaccination of catfish fry in catfish production systems. Results for mutant strains *Ei*Δ*sdhC*, *Ei*Δ*mdh* and *Ei*Δ*gcvP* were similar to our previous study that evaluated vaccine efficacy of *E. ictaluri sdhC*, *mdh*, and *gcvP* transposon insertion mutants [21]. In our previous study, *sdhC* and *mdh* insertion mutants gave 100% protection against *E. ictaluri* infection, and a *gcvP* insertion mutant gave 89.15% survival in catfish fingerlings. Our current results with deletion mutants show that attenuation is not due to polar effects of the insertion mutations. The deletion mutants have an additional advantage in that they do not carry antibiotic resistance genes. The current study is the first to report vaccine efficacy of *E. ictaluri Ei*Δ*glyA* and *Ei*Δ*frdA* mutants; both provided significant protection against mortalities by immersion vaccination.

We also evaluated vaccine efficacy of our candidate mutant strains using a more sensitive measure than percent survival; namely, we evaluated the ability of the mutant strains to prevent invasion of virulent *E. ictaluri* as monitored using BLI. Vaccination in this trial was by injection, which is not a practical route of vaccination for commercial catfish production, but it does allow accurate vaccine dose delivery. Protection results by injection vaccination were very similar to results obtained by immersion vaccination, except that *Ei*Δ*glyA* vaccination provided better protection by immersion vaccination than injection (Fig. 4). It is possible that immersion vaccination using *Ei*Δ*glyA* may activate mucosal immunity better, preventing wild type *E. ictaluri* septicemia. We saw the opposite trend when fish were vaccinated with the *Ei*Δ*gcvP* mutant, which protects fish better when vaccination is applied by injection rather than immersion.

Succinate dehydrogenase (SDH) is part of the aerobic respiratory chain in the TCA cycle, oxidizing succinate to fumarate while reducing ubiquinone to ubiquinol [22]. It is closely related to fumarate reductase, which catalyzes the reverse reaction. Succinate dehydrogenase and fumarate reductase can replace each other [22], [23]. Although SdhC has similar function, hydrophobicity, and protein size to the membrane-binding subunit fumarate reductase (FrdC), *sdhC* and *frdC* do not share significant sequence identity [24]. The organic acids formate and succinate have a protective effect in stationary phase cells against killing effects of antimicrobial peptide BPI, which appears to disrupt the bacterial respiratory chain [25]. Maintenance of protective levels of formate and succinate requires the activity of formate dehydrogenase and succinate dehydrogenase, respectively.

In *E. coli* and *Salmonella*, succinate dehydrogenase is known to contribute to pathogenicity. Recently, it was shown that a full TCA cycle is required for *Salmonella enterica* virulence, and a *sdhDCA* mutant is attenuated in an oral mouse infection model [26], which is similar to our finding. In *Helicobacter pylori*, fumarate reductase was found to be essential for colonization of mouse gastric mucosa [27]. In *Salmonella enterica*, deletion of *sdhCDA* caused partial attenuation, and complete attenuation was achieved when both *sdhCDA* and *frdABCD* were deleted [28]. Our results indicated that deletion of only the *E. ictaluri sdhC* gene and deletion of only *frdA* resulted in full attenuation in catfish fingerlings. However, our previous results showed that catfish fry are more sensitive to *E. ictaluri* than catfish fingerlings (unpublished data), so further testing in catfish fry is warranted. Regardless, the data show that succinate dehydrogenase and fumarate reductase play an important role in pathogenesis. The other mutant that was tested in this study was *mdh*, which encodes malate dehydrogenase. Our results show that *mdh* is also important in *E. ictaluri* virulence, which was consistent with findings in *Salmonella* using the mouse oral challenge model, where a *mdh* mutant was found to be highly attenuated [26].

The glycine cleavage system is a loosely associated four subunit enzyme complex that catalyzes the reversible oxidation of glycine to form 5, and 10-methylenetetrahydrofolate, which serves as a one carbon donor. It is one of two sources of C1 units; serine hydroxymethyltransferase is another source, and it is considered a more important source. Expression of the glycine cleavage enzyme system is induced by glycine [29], [30], and *gcv* mutants are unable to use glycine as a C1 source and excrete glycine [31]. We have previously shown that *E. ictaluri gcvP* is required for virulence [14]. This is the first report that *glyA* is required for *E. ictaluri*, virulence, and to our knowledge, this is the first report that serine hydroxymethyltransferase is associated with virulence in any bacterial species.

Although BLI for real-time monitoring of *E. ictaluri* infection in live fish was shown by our group [17], this is the first time we report the use of BLI to quantify the degree of *E. ictaluri* attenuation in channel catfish. It appears that BLI could be used for vaccine evaluation by using a relatively low number of fish (four fish in this work). Also, use of BLI provides a more sensitive measure of vaccine protection than percent mortalities.

In summary, our results showed that the *EiΔsdhC*, *EiΔmdh*, *EiΔfrdA*, *EiΔgcvP*, and *EiΔglyA* mutants were significantly attenuated and provided protection against ESC under controlled laboratory conditions. Thus, *EiΔsdhC*, *EiΔmdh*, *EiΔfrdA*, and *EiΔgcvP* mutants have potential for use as live attenuated vaccines for catfish fingerlings. The *E. ictaluri ΔglyA* mutant was found to be incapable of persisting in catfish when injected, which might be the reason for lower protection than when it is used in immersion vaccination. Based on these results, testing of these vaccine candidates in catfish fry is warranted.
